# Brucellosis in Guangdong Province, People’s Republic of China, 2005–2010

**DOI:** 10.3201/eid1905.120146

**Published:** 2013-05

**Authors:** Jing-diao Chen, Chang-Wen Ke, Xiaoling Deng, Shu Jiang, Wenjia Liang, Bi-Xia Ke, Baisheng Li, Hailing Tan, Meizhen Liu

**Affiliations:** Center for Disease Control and Prevention, Guangzhou, China

**Keywords:** brucellosis, reemerging, exposure, Brucella melitensis, Brucella suis, biovar, Guangdong Province, China, zoonoses, bacteria

**To the Editor:** Brucellosis is one of the most prevalent zoonotic diseases in the world. It is principally an animal disease, but globally, >500,000 human cases are reported each year (*1*). Transmission to humans occurs primarily through contact with infected animals and consumption of contaminated food (*2*,*3*). Persons with occupational exposure are at highest risk for brucellosis, in particular those performing husbandry activities, butchering, and livestock trading (*4*,*5*).

Although brucellosis has been eradicated from many industrialized countries, new foci of disease continually appear, particularly in parts of Asia (*6*–*8*). In China, 160,214 brucellosis cases were reported during 2005–2010; 90% of them occurred in 6 northern agricultural provinces: Neimenggu, Shanxi, Heilongjiang, Hebei, Jilin, and Shaanxi. Livestock, such as goats, cattle, and pigs, are the main infectious source. However, factors such as the rapid movement of people from northern to southern China, increased livestock trading, and lack of livestock quarantine mean that infected livestock or their products readily traverse provincial borders and transmit disease to persons who have no direct contact with livestock.

With an illness rate of <0.01 cases/100,000 population, Guangdong Province in southern China is one of the areas in China with the lowest incidence of brucellosis (*9*), but incidence is increasing. During 1955–2004, Guangdong Province recorded 51 confirmed cases of brucellosis; however, during 2005–2010, 112 cases were reported. All reported cases had typical clinical characteristics, including undulant fever, night sweats, chills, and weakness; some cases were associated with encephalitis, meningitis, and arthritis. Of the 112 reported cases during 2005–2010, 105 were laboratory confirmed: 61 by culture (55 from blood culture, 3 from bone marrow, and 1 each from joint fluid, cerebrospinal fluid, and a vertebrae disc abscess); and 44 by serum agglutination test (SAT; single titer >400). The male:female ratio among these patients was 66:46. The age ranges were similar by sex; male patients were 18–71 (median 47) years of age, and female patients were 20–70 (median 43) years of age.

The first 3 cases of brucellosis in 2005 were reported in Shenzhen in Guangdong Province. One case was culture confirmed by clinical laboratory, and the isolate was identified as *Brucella melitensis* biovar 3 by SAT and phage biotyping. The other 2 cases were in dairy farm workers; their infections were laboratory confirmed by SAT but could not be identified by biovar. Since 2005, more cities in Guangdong have reported brucellosis cases (Figure, Appendix). The Pearl River Delta region reported 100 cases: 48 in Guangzhou, 27 in Shenzhen, 7 in Zhongshan, 6 in Foshan, 6 in Jiangmen, 4 in Zhuhai, and 2 in Dongguan. Only 12 cases were reported from undeveloped rural areas in Guangdong: 5 in Zhaoqing, 2 in Yangjiang, and 1 each in Huizhou, Qingyuan, Meizhou, Maoming, and Yunfu. 

**Figure:**
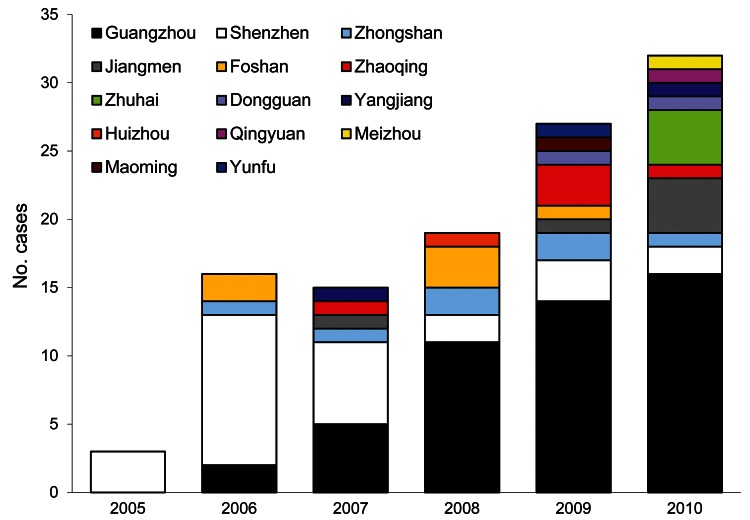
Reported cases of brucellosis, by city, Guandong Province, China, 2005–2010.

A total of 42 *Brucella* isolates were cultured during 2005–2009, and all were identified as *B. melitensis* biovar 3. However, of 19 *Brucella* isolates cultured during 2010, a total of 13 were identified as *B. melitensis* biovar 3, 4 as *B. melitensis* biovar 1, and 2 as *B. suis* biovar 3. These results indicate a shift in species and biovar for *Brucella* spp. circulating in China. 

We conducted a retrospective epidemiologic investigation of the 112 brucellosis cases reported during 2005–2010 to identify the vehicles and sources of infection. Among the cases identified, 33 (29.46%) patients had occupational exposure history: 13 were pig or goat butchers, 12 dairy farmers, 5 animal market workers in charge of leading the animals to and from transportation, and 3 mutton and pork sellers in wet markets. The remaining 79 (70.54%) cases were in patients who denied having contact with living animals. Among these patients were retired persons, housekeeping matrons, teachers, doctors, white collar workers, and the unemployed. However, 17 of these patients recalled having purchased or handled goat placenta to be prepared for home consumption or having eaten goat products through barbequing or hot pot. The other 62 could not remember if they had contacted with livestock or their products. These findings indicate that nonoccupational exposure may pose a risk for brucellosis infection among persons who handle fresh meat and meat products for home cooking.

In conclusion, Guangdong Province has become an emerging foci for brucellosis in China. The species and biovars of *Brucella* spp. circulating in this region are changing, and many persons are infected by nonoccupational exposure. Measures need to be taken by central and provincial governments to address these issues and prevent epidemics of brucellosis in humans.
